# Reports of elevated cognitive decline in autistic adults are linked to high anticholinergic potency

**DOI:** 10.1002/alz70857_100513

**Published:** 2025-12-24

**Authors:** Gregory L Wallace, Goldie A McQuaid, Sean C Duane, Neha Ahmed, Annissa DeSilva, Claire B Klein, Laura Klinger, Rebecca A Charlton, Nancy R Lee

**Affiliations:** ^1^ The George Washington University, Washington D.C., DC, USA; ^2^ George Mason University, Fairfax, VA, USA; ^3^ The George Washington University, Washington, DC, USA; ^4^ University of North Carolina, Chapel Hill, Chapel Hill, NC, USA; ^5^ University of North Carolina, Chapel Hill, NC, USA; ^6^ Goldsmiths, University of London, London, United Kingdom; ^7^ Drexel University, Philadelphia, PA, USA

## Abstract

**Background:**

Emerging evidence from healthcare claims data indicates that autistic adults receive dementia‐related diagnoses at increased rates compared to the general population. Nevertheless, prospective data, including dementia risk screeners, have rarely been collected to directly evaluate risk for cognitive decline among autistic adults. Given high rates of polypharmacy, autistic adults likely experience high anticholinergic burden, which is linked to dementia risk in the general population. The current study uses screeners to assess risk for cognitive decline, documents anticholinergic burden based on reported medication use, and links cognitive decline and anticholinergic burden in two relatively large samples of autistic adults.

**Method:**

Sample 1 includes 210 “independent” autistic adults (age range=42‐81yrs, M=56yrs; 58% female‐assigned‐sex‐at‐birth). Sample 2 includes 500 “dependent” autistic adults (age range=18‐68yrs, M=31yrs; 20% female‐assigned‐sex‐at‐birth). Both samples were recruited via SPARK, a research participant registry of autistic people and their caregivers (“independent” vs. “dependent” refers to the autistic adults’ ability to independently consent). Cognitive decline was assessed using the self‐rated AD8 among independent autistic adults and via the caregiver‐rated Dementia Screening Questionnaire for Individuals with Intellectual Disabilities (DSQIID) for “dependent” autistic adults. The CRIDECO Anticholinergic Load Scale/CALS was used to code medications for anticholinergic potency.

**Result:**

30% of independent autistic adults endorsed 2 or more symptoms, thus they “screened positive” for cognitive decline on the AD8, and 10% of dependent autistic adults had caregiver ratings indicating 1 or more memory/communication declines specifically (an index of cognitive decline) and 3% “screened positive” using the more conservative 20+ DSQIID cutoff score. Anticholinergic medication use was high in both groups, with >63% of individuals in both samples taking 1 or more anticholinergic medications. Finally, dimensional ratings of cognitive decline were significantly associated with anticholinergic potency in both samples after accounting for the effects of age and assigned‐sex‐at‐birth (independent: B=0.16, R^2^Change=0.02; dependent: B=0.18, R^2^Change=0.03; *p*s < .01).

**Conclusion:**

Ratings from dementia screeners dovetail with healthcare claims data by revealing relatively common reports of cognitive decline in autistic adults. Furthermore, among autistic adults, anticholinergic medication use is high, and increased anticholinergic potency is linked to reports of cognitive decline, often at younger ages than in the general population.

